# Fabrication of a novel graphene oxide based magnetic nanocomposite and its usage as a highly effectual catalyst for the construction of *N*,*N*′-alkylidene bisamides†

**DOI:** 10.1039/d4ra04136d

**Published:** 2024-08-13

**Authors:** Abdolkarim Zare, Marziyeh Barzegar, Esmael Rostami, Ahmad Reza Moosavi-Zare

**Affiliations:** a Department of Chemistry, Faculty of Nano and Bio Science and Technology, Persian Gulf University Bushehr 75169 Iran a.zare@pgu.ac.ir abdolkarimzare@yahoo.com; b Department of Chemistry, Payame Noor University PO Box 19395-4697 Tehran Iran barzegar.marziyeh@yahoo.com; c Department of Chemical Engineering, Hamedan University of Technology Hamedan 65155 Iran; d Chemistry Department, College of Sciences, Shiraz University Shiraz 71946-84795 Iran

## Abstract

At first, a novel graphene oxide-based magnetic nanocomposite namely Si-propyl-functionalized *N*^1^,*N*^1^,*N*^2^,*N*^2^-tetramethylethylenediamine-*N*^1^,*N*^2^-diium hydrogen sulfate anchored to graphene oxide-supported Fe_3_O_4_ (nano-[GO@Fe_3_O_4_@R-NHMe_2_][HSO_4_]) was fabricated. After full characterization of the nanocomposite, its catalytic performance was examined for the solvent-free construction of *N*,*N*′-alkylidene bisamides from aryl aldehydes (1 eq.) and primary aromatic and aliphatic amides (2 eq.), in which the products were acquired in short times (15–30 min) and high to excellent yields (89–98%). Nano-[GO@Fe_3_O_4_@R-NHMe_2_][HSO_4_] could be magnetically isolated form the reaction medium, and reused three times without remarkable loss of catalytic activity.

## Abstract 2

A novel graphene oxide-based magnetic nanocomposite namely nano-[GO@Fe_3_O_4_@R-NHMe_2_][HSO_4_] was fabricated, characterized and applied in the solvent-free construction of *N*,*N*′-alkylidene bisamides from aryl aldehydes (1 eq.) and primary amides (2 eq.).

## Graphical abstract

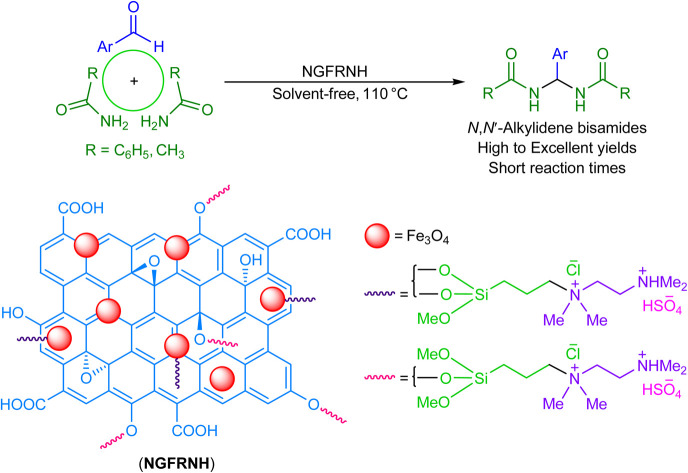

## 1. Introduction

Graphene oxide (GO) is made from flat sheets with hydroxyl, epoxide and carboxylic acid groups; in these sheets, carbon atoms with sp^2^ and sp^3^ hybridization are placed into a honeycomb network. Besides the unique characteristics of GO such as high structural strength, appropriate durability (chemical and thermal), safety, high adsorption capacity, high hydrophilic nature, high thermal conductivity and suitable mechanical properties, it can be readily functionalized using inorganic (magnetic/non-magnetic) and organic components to fabricate GO derivatives for different uses.^1–22^ For example, GO and its functionalized derivatives (magnetic nanocomposites, *etc.*) have been used for treatment of hazardous environmental contaminants,^1^ targeted delivery of quercetin to cancer cells,^2^ sustainable water purification,^3^ extracting and determining metoprolol in exhaled breath condensate,^4^ removing dyes from wastewater^5^ and cancer therapy.^6^ They have been also applied as adsorbents,^7^ heat exchangers,^8^ bioinks for three-dimensional mesenchymal stem cell printing^9^ and biosensors.^10^ In organic synthesis, GO and its derivatives have been utilized as efficacious catalysts.^11–22^

The high importance and numerous applications of magnetic nanomaterials have been reported in the literature.^3–7,17–23^ Some advantages of these materials include safety, suitable thermic and chemical durability, easy detaching from the process reactor, non-corrosiveness, effectiveness and aptitude to graft with diverse inorganic and organic components for a wide range of usages.^3–7,17–23^ It is worth noting that in magnetic nanocomposites based on GO, the advantages of magnetic nanomaterials and graphene oxide have been studied.

A valuable, useful, advantageous and applicable protocol, which has been extensively utilized for the construction of numerous organic substances, is the use of solvent-free conditions.^24–28^ Utilization of this protocol not only is in accordance with the principles of green chemistry, but it can also lead to cleaner reaction medium, easier workup, increasing yield, decrement of reactor size and decreasing energy consumption, time and cost.^25^

Bisamide scaffolds exist in the structure of numerous industrial and bioactive compounds.^29–37^ For instance, these compounds have been applied for selective dye uptake,^29^ selective detection of metal ions,^30^ removal of Hg^2+^ and Pb^2+^ ions^31^ and ampere sensing.^32^ Moreover, they have been utilized as additives to control formation of methane hydrate for gas storage and flow assurance,^33^ highly stable MRI contrast,^34^ tyrosinase inhibitors,^35^ antitumor^36^ and antiviral^37^ agents. A group of these compounds is the *N*,*N*′-alkylidene bisamides, which have been manufactured through the condensation of aryl aldehydes (1 eq.) with primary amides (2 eq.) using a catalyst.^38–47^

Having the above issues in mind, developing a novel graphene oxide-based magnetic nanocomposite as a catalyst for the construction of *N*,*N*′-alkylidene bisamides can be valuable and desirable. Herein, we have developed Si-propyl-functionalized *N*^1^,*N*^1^,*N*^2^,*N*^2^-tetramethylethylenediamine-*N*^1^,*N*^2^-diium hydrogen sulfate anchored to graphene oxide-supported Fe_3_O_4_ (nano-[GO@Fe_3_O_4_@R-NHMe_2_][HSO_4_] or NGFRNH) to catalyze the construction of *N*,*N*′-alkylidene bisamides.

## 2. Experimental

### 2.1. Materials and instruments

The details of the materials and instruments used have been described in the ESI.†

### 2.2. Fabrication of NGFRNH

**Scheme 1:**
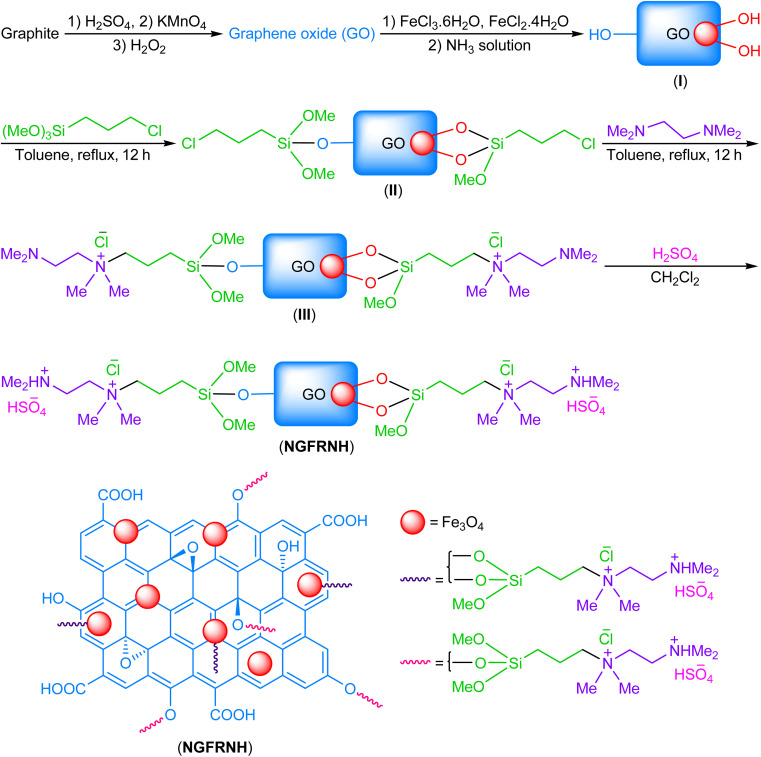
The fabrication of NGFRNH.

*Note*: Before each stage, the reaction mixture was irradiated with ultrasound waves to disperse it.

GO and GO@Fe_3_O_4_ (I) were constructed through the reported protocols.^48,49^ (3-Chloropropyl)trimethoxysilane (3 mmol, 0.596 g) and toluene (30 mL) were added to I (1.5 g), and stirred in reflux conditions for 12 h; the solid was magnetically isolated, washed with toluene (2 × 5 mL), and dried under vacuum (at 100 °C) to furnish II. Thereupon, *N*^1^,*N*^1^,*N*^2^,*N*^2^-tetramethylethylenediamine (3 mmol, 0.349 g) and compound II were stirred and refluxed in toluene (30 mL) for 12 h; the solid was separated by an external magnet, washed with toluene (2 × 5 mL), and dried under vacuum (at 100 °C) to produce III. Lastly, H_2_SO_4_ (3 mmol, 0.16 mL) was gradually added to III in CH_2_Cl_2_ (20 mL) at ambient temperature, and stirred for 5 h at the same temperature and 2 h under reflux conditions; the solid was magnetically separated, washed by CH_2_Cl_2_ (2 × 5 mL), and dried at 100 °C (under vacuum) to fabricate NGFRNH (Scheme 1).

### 2.3. The construction of *N*,*N*′-alkylidene bisamides (general protocol)

A mixture of an aldehyde (0.5 mmol), amide (1 mmol) and NGFRNH (0.040 g) in a reaction vessel was strongly stirred at 110 °C using a glass rod. After observing consumption of the aldehyde and amide by TLC, the mixture was cooled to ambient temperature, warm EtOAc (10 mL) was added to it, and stirred for 1 min; then, NGFRNH was magnetically isolated (this action was done two times); the recycled NGFRNH was washed with EtOAc (2 × 3 mL), dried and used for next run. The acquired solutions after the double extraction of the product were collected and distilled; the remaining solid was recrystallized from ethanol (95%) to construct the pure bisamide.

*Note*: Selected original spectra of the bisamides are provided in the ESI.†

### 2.4. Selected spectral data of the constructed bisamides

#### Bisamide 3

^1^H NMR (300 MHz, DMSO-*d*_6_): *δ* (ppm) 7.20 (t, *J* = 7.1 Hz, 1H, methine CH), 7.50–7.62 (m, 6H, H_Ar_), 7.73 (t, *J* = 7.9 Hz, 1H, H_Ar_), 8.00 (d, *J* = 7.7 Hz, 5H, H_Ar_), 8.23 (d, *J* = 8.0 Hz, 1H, H_Ar_), 8.42 (s, 1H, H_Ar_), 9.32 (d, *J* = 7.2 Hz, 2H, 2NH); ^13^C NMR (75 MHz, DMSO-*d*_6_): *δ* (ppm) 59.1, 121.9, 123.3, 128.1, 128.8, 130.4, 132.2, 134.1, 134.2, 142.9, 148.3, 166.5.

#### Bisamide 8

^1^H NMR (300 MHz, DMSO-*d*_6_): *δ* (ppm) 7.10 (t, *J* = 6.6 Hz, 1H, methine CH), 7.51–7.60 (m, 6H, H_Ar_), 7.85 (t, *J* = 8.5 Hz, 2H, H_Ar_), 7.97 (d, *J* = 7.6 Hz, 4H, H_Ar_), 8.24 (s, 1H, H_Ar_), 9.27 (d, *J* = 6.6 Hz, 2H, 2NH); ^13^C NMR (75 MHz, DMSO-*d*_6_): *δ* (ppm) 58.7, 124.4, 124.6, 128.2, 128.8, 132.0, 132.2, 132.8, 134.0, 141.8, 148.0, 166.5. Mass: *m*/*z* 409 (M^+^).

#### Bisamide 10

^1^H NMR (300 MHz, DMSO-*d*_6_): *δ* (ppm) 7.12 (t, *J* = 7.5 Hz, 1H, methine CH), 7.25 (t, *J* = 8.8 Hz, 2H, H_Ar_), 7.49–7.61 (m, 8H, H_Ar_), 7.98 (d, *J* = 7.0 Hz, 4H, H_Ar_), 9.12 (d, *J* = 7.6 Hz, 2H, 2NH); ^13^C NMR (75 MHz, DMSO-*d*_6_): *δ* (ppm) 58.9, 115.4, 115.7, 128.0, 128.8, 129.1, 129.2, 132.1, 134.3, 137.1, 160.6, 163.8, 166.2.

## 3. Results and discussion

### 3.1. Characterization of NGFRNH

At first, GO was produced by oxidation of graphite using a rectified Hummers' protocol. Then, Fe_3_O_4_ nanoparticles was supported on GO nanosheets using co-precipitation method to synthesize GO@Fe_3_O_4_. In continue, GO@Fe_3_O_4_ was functionalized by (3-chloropropyl)trimethoxysilane, *N*^1^,*N*^1^,*N*^2^,*N*^2^-tetramethylethylenediamine and sulfuric acid to fabricate nano-[GO@Fe_3_O_4_@R-NHMe_2_][HSO_4_] (NGFRNH) as a novel graphene oxide based magnetic nanocomposite. The structure of NGFRNH was proposed on basis of the reported structures for this category of materials.^19,20,49^ Energy-dispersive X-ray spectroscopy (EDX), elemental mapping, field emission scanning electron microscopy (FE-SEM), FT-IR, X-ray diffraction (XRD), thermogravimetric (TG), derivative thermogravimetry (DTG) and vibrating-sample magnetometery (VSM) analyses were used to characterize the nanocomposite.

**Fig. 1:**
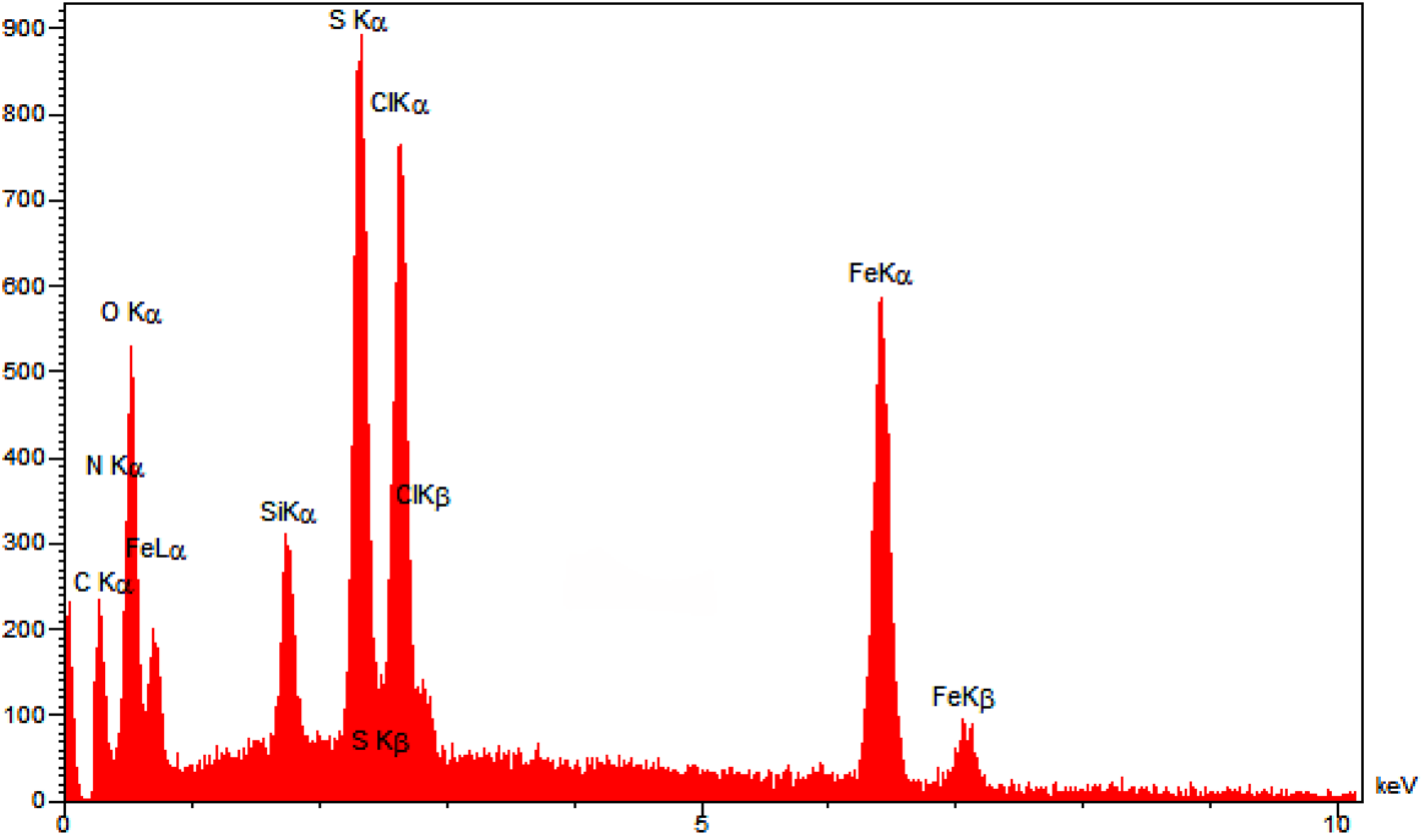
The EDX analysis of NGFRNH.

**Fig. 2:**
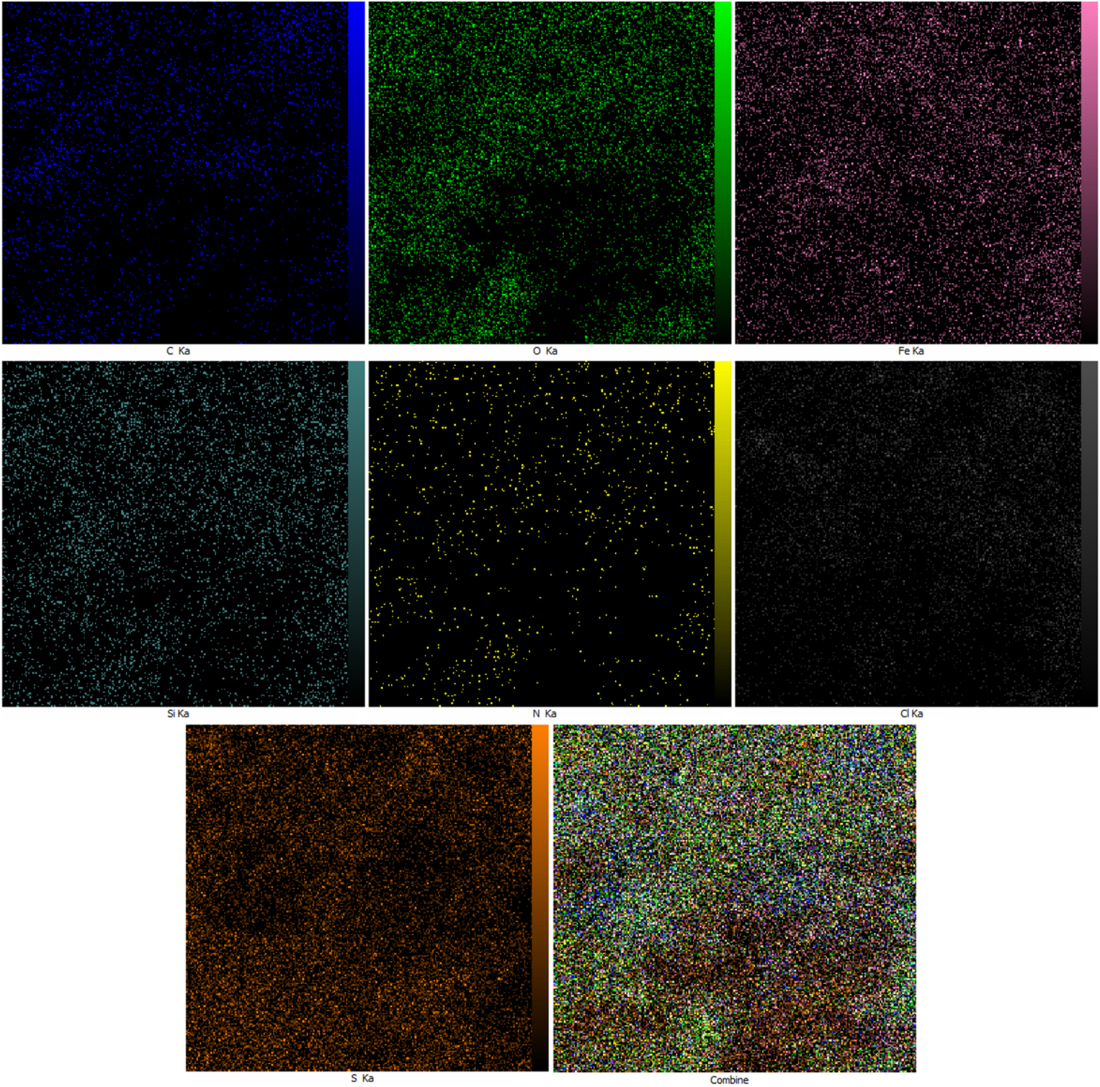
The elemental mapping images of NGFRNH.

The EDX (Fig. 1) and elemental mapping (Fig. 2) analyses of nano-[GO@Fe_3_O_4_@R-NHMe_2_][HSO_4_] showed carbon, which is pertained to GO and the organic moiety anchored to Fe_3_O_4_. The analyses indicated oxygen, which is ascribed to GO, Fe_3_O_4_ and HSO_4_^−^. Observation of the peak related to iron in the EDX spectrum, and observing iron in the elemental mapping images confirmed existing Fe_3_O_4_ in the nanocomposite structure. Both analyses verified existing silicon, which is belong to Si-propyl-functionalized *N*^1^,*N*^1^,*N*^2^,*N*^2^-tetramethylethylenediamine-*N*^1^,*N*^2^-diium moiety. The peak assigned to nitrogen of *N*^1^,*N*^1^,*N*^2^,*N*^2^-tetramethylethylenediamine-*N*^1^,*N*^2^-diium component was observed in the EDX spectrum; nitrogen was also seen in the elemental mapping analysis. The chlorine (related to Cl^−^) was observed in both analyses. Observation of S in the EDX and elemental mapping analyses approved existing HSO_4_^−^ in the structure of NGFRNH. Furthermore, the elemental mapping images demonstrate good distribution of the elements in the nanocomposite surface.

**Fig. 3:**
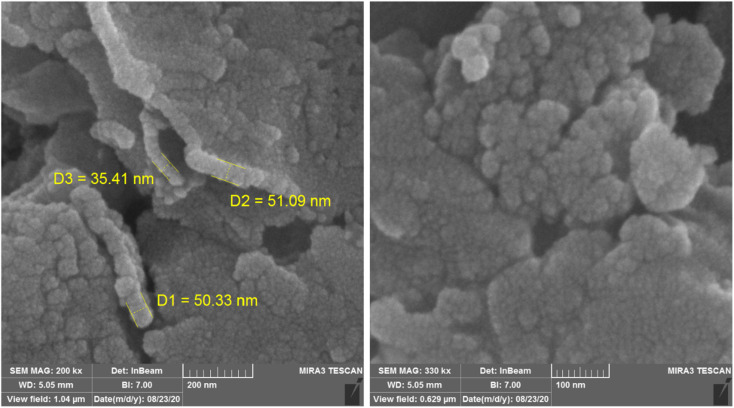
The FE-SEM pictures of the nanocomposite.

Fig. 3 illustrates the FE-SEM pictures of NGFRNH; the pictures showed nanosheets of GO with diameter of 35.4, 50.3, 51.1 nm, *etc.* and crumpled structure in their edges, and also the nanoparticles of the functionalized Fe_3_O_4_ supported on GO.

**Fig. 4:**
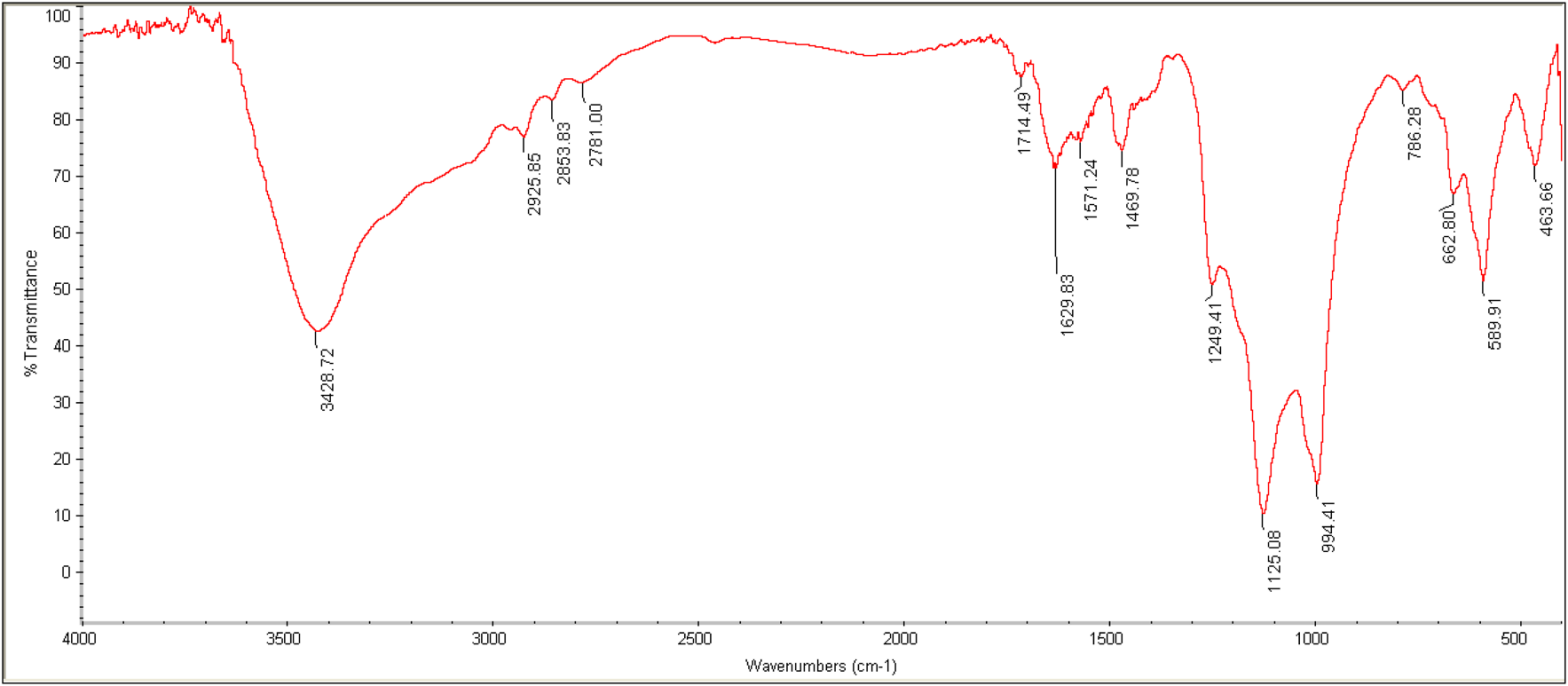
The FT-IR spectrum of NGFRNH.

The FT-IR spectrum of nano-[GO@Fe_3_O_4_@R-NHMe_2_][HSO_4_] is represented in Fig. 4, and the interpretation of the spectrum is given in Table 1. The spectrum showed the peaks related to all bonds and functional groups presented in the nanocomposite structure (graphene oxide, Fe_3_O_4_, OSi-R′-NHMe_2_ and HSO_4_^−^); thus, the spectrum confirmed successful fabrication of the catalyst, *i.e.* supporting Fe_3_O_4_ on GO to produce GO@Fe_3_O_4_, and functionalization of GO@Fe_3_O_4_ by the organic component and HSO_4_^−^.

**Table 1:** The results on interpreting the FT-IR spectrum of NGFRNH

Peak (cm^−1^)	Bond or functional group
464	Si–O (rocking)
590	Fe–O (stretching vibration)
1125	SO_2_ of HSO_4_^−^ (symmetric stretching)
1249	SO_2_ of HSO_4_^−^ (asymmetric stretching)
1470	Aliphatic C–H (bending)
1630	C=C of GO (stretching vibration)
1714	C=O of GO (stretching vibration)
2926	Aliphatic C–H (stretching vibration)
∼2570–3630	OH groups of HSO_4_^−^ and GO (stretching)

**Fig. 5:**
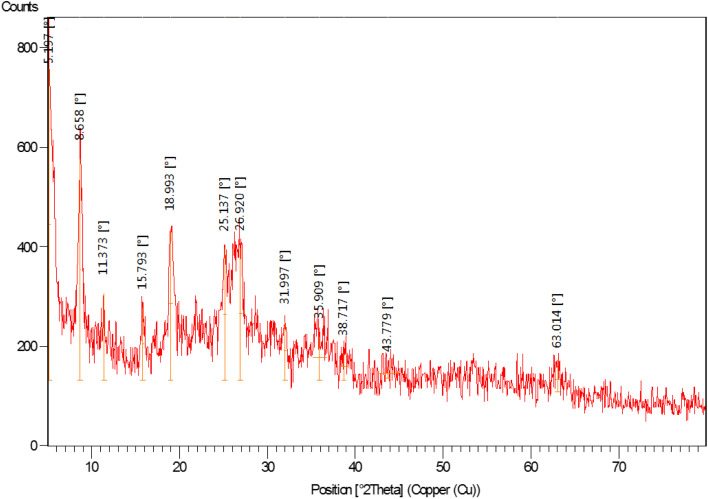
The XRD spectrum of NGFRNH.

The XRD pattern of nano-[GO@Fe_3_O_4_@R-NHMe_2_][HSO_4_] is displayed in Fig. 5. The peak located at 11.37° can be related to GO; the low intensity of the peak is because of supporting Fe_3_O_4_ on GO nanosheets and also functionalization by Si-R′-NHMe_2_ and hydrogen sulfate. The diffraction lines appeared at 31.99, 35.91, 38.72, 43.78, 53.39, 58.38 and 63.01° verified existing Fe_3_O_4_ (a cubic spinel form) in the nanocomposite structure, and consequently, successful supporting Fe_3_O_4_ on GO nanosheets. The other data obtained from the XRD pattern, such as FWHM (width at half maximum), interplanar distance, relative intensity of the peaks and crystalline sizes of the particles, are illustrated in Table 2; the crystalline sizes, which were calculated by Debye–Scherrer equation, were in the range of 3.63–54.13 nm, and are in acceptable compliance with the sizes gained from the FE-SEM analysis (Fig. 3).

**Table 2:** The XRD data of for NGFRNH

2*θ* (°)	FWHM (°)	Interplanar distance (nm)	Rel. int. (%)	Crystalline size (nm)
5.197	0.2952	1.7006	100.00	26.96
8.658	0.1968	1.0213	71.04	40.52
11.373	0.1476	0.7780	27.83	54.13
15.793	0.2952	0.5611	23.25	27.19
18.993	0.3936	0.4673	48.69	20.48
25.137	0.3936	0.3543	42.09	20.70
26.920	0.3936	0.3312	42.21	20.77
31.997	0.3936	0.2797	18.55	21.01
35.909	1.5744	0.2500	13.94	5.30
38.717	0.9840	0.2326	8.49	8.56
43.779	2.3616	0.2068	2.83	3.63
63.014	0.5904	0.1475	8.53	15.79

**Fig. 6:**
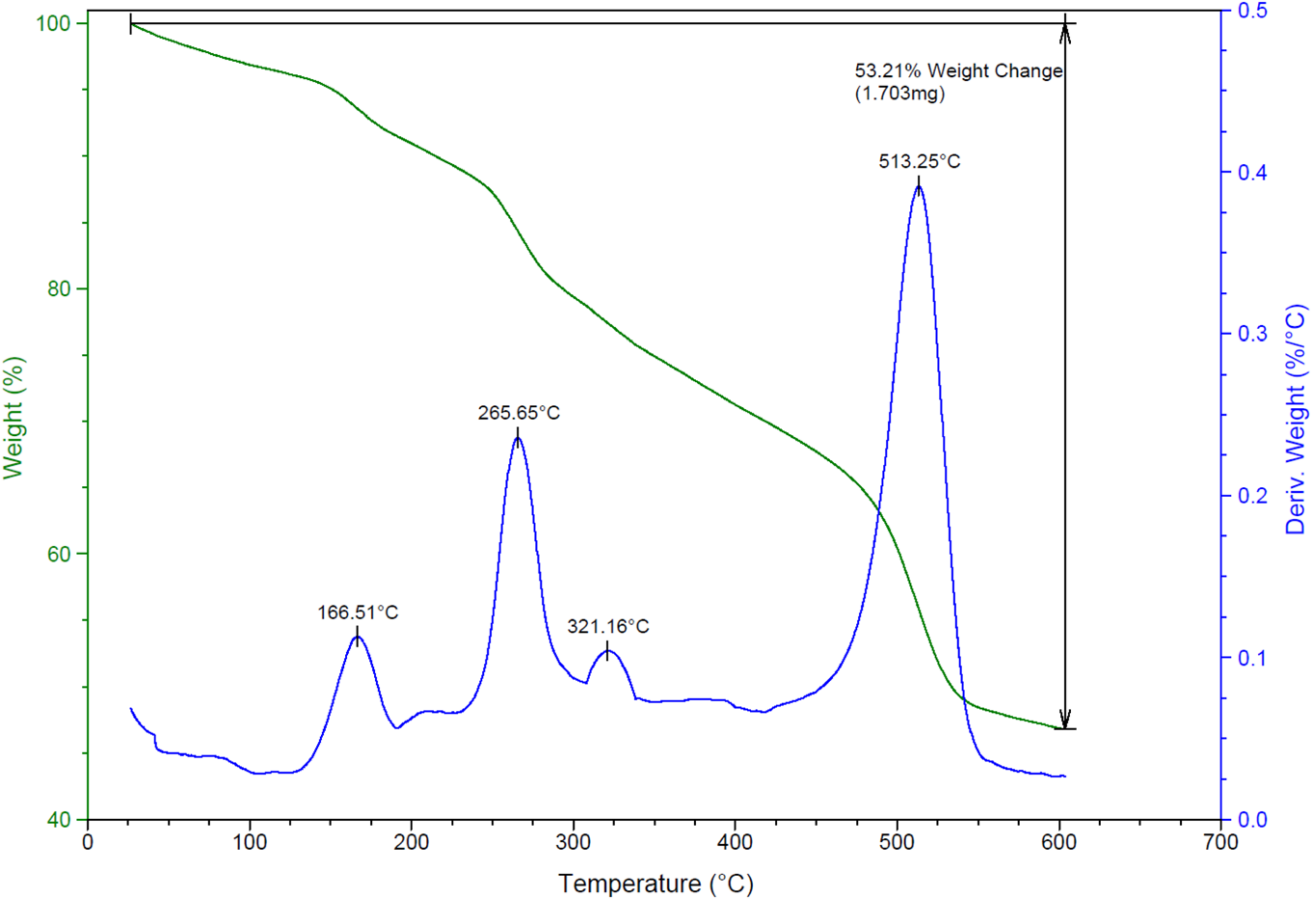
The TG and DTG curves of NGFRNH.

Thermal durability of NGFRNH was determined by TG and DTG analyses (Fig. 6); the corresponding diagrams demonstrate weight losing in three stages. The weight loss occurred up to 175 °C (with *T*_max_ at 166.5 °C in the DTG diagram) can be related to thermal desorption of water and solvents adsorbed on the nanocomposite surface. The second and third stages of the weight losing which took place at 175–320 °C (with *T*_max_ at 265.7 °C in the DTG diagram) and 320–600 °C (with *T*_max_ at 513.2 °C in the DTG diagram) can be due to the decomposition of oxygen-containing groups in GO (carboxylic acid, hydroxyl and epoxide) and the organic constitute grafted with GO@Fe_3_O_4_ (*i.e.* Si-R′-NHMe_2_). The weight loss after 470 °C is related to decomposition of GO nanosheets.

**Fig. 7:**
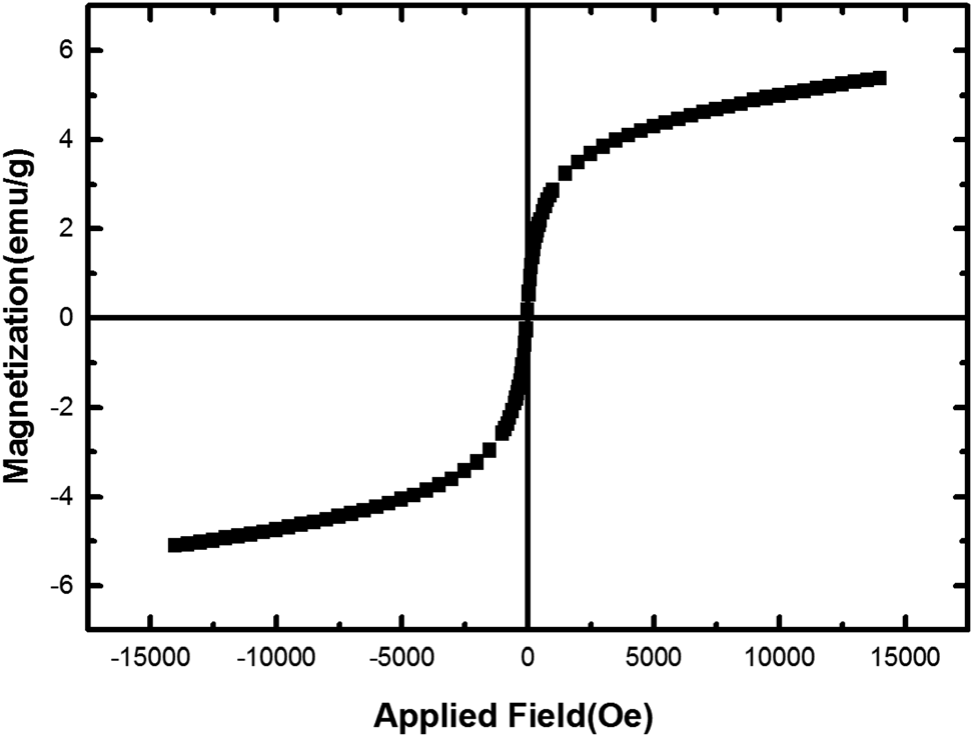
The VSM diagram of the magnetic nanocomposite.

Magnetic behavior of nano-[GO@Fe_3_O_4_@R-NHMe_2_][HSO_4_] was studied by VSM analysis; Fig. 7 depicts the analysis result. Considering the VSM diagram, saturation magnetization (*M*_s_) of the nanocomposite was ∼5.4 emu g^−1^. Lower *M*_s_ of NGFRNH compared to Fe_3_O_4_ is due to supporting Fe_3_O_4_ on graphene nanosheets and functionalization with the organic component and hydrogen sulfate. Nevertheless, NGFRNH had sufficient magnetic property to recycle from the reaction mixture by an external magnet.

### 3.2. Application of NGFRNH as catalyst for manufacturing *N*,*N*′-alkylidene bisamides

**Scheme 2:**
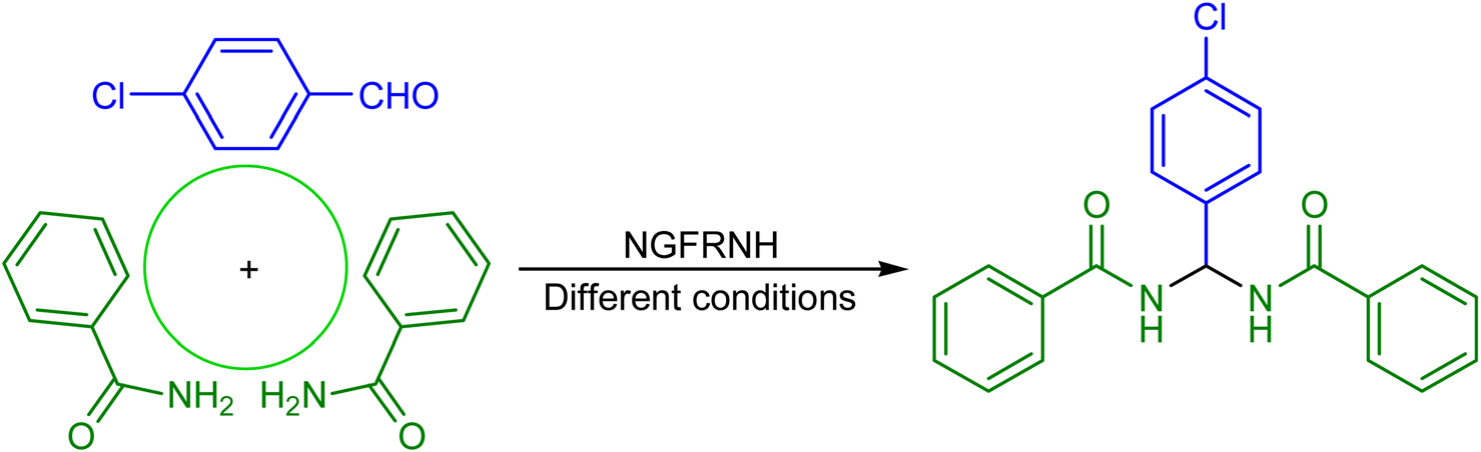
The model reaction to acquire the best conditions.

Investigating catalytic property of nano-[GO@Fe_3_O_4_@R-NHMe_2_][HSO_4_] was done on the construction of *N*,*N*′-alkylidene bisamides from aryl aldehydes and primary amides. In this regard, the condensation of 4-chlorobenzaldehyde (0.5 mmol) and benzamide (1 mmol) was tested using 0.035, 0.040 and 0.045 g of NGFRNH at 90, 100, 110 and 115 °C in the absence of solvent; Scheme 2 illustrates the model reaction, and Table 3 indicates the obtained results. The best results were attained when the reaction was performed using 0.040 g of the nanocomposite at 110 °C (entry 2); so, 0.040 g was chosen as the optimal catalyst dosage, and 110 °C was selected as the optimized temperature. To compare catalytic efficacy of nano-[GO@Fe_3_O_4_@R-NHMe_2_][HSO_4_] with the precursors used for its synthesis, and determine the role of graphene oxide, the model reaction was examined without catalyst and also in the presence of the precursors (GO, II and III) under identical conditions. As Table 3 exemplifies, these conditions were not efficient, and afforded low or moderate yields of product 6 (entries 7–10). Thus, our plan to design nano-[GO@Fe_3_O_4_@R-NHMe_2_][HSO_4_] as catalyst for the fabrication of *N*,*N*′-alkylidene bisamides was logical. Furthermore, considering the results acquired in entries 7 and 8, GO role was not only as a support, but also it could act as a co-catalyst. In another study, the gram scale synthesis of product 6 was studied; for this purpose, 5 mmol (0.703 g) of 4-chlorobenzaldehyde was reacted with 10 mmol (1.211 g) of benzamide in the presence of 0.400 g of NGFRNH at 110 °C, in which the bisamide was obtained in 93% after 20 min.

**Table 3:** Optimization of the reaction conditions

Entry	Catalyst	Catalyst amount (g)	Temp. (°C)	Time (min)	Yield^a^ (%)
1	NGFRNH	0.035	110	25	93
2	NGFRNH	0.040	110	15	98
3	NGFRNH	0.045	110	15	98
4	NGFRNH	0.040	90	35	76
5	NGFRNH	0.040	100	25	91
6	NGFRNH	0.040	115	15	98
7	—	—	110	15	<10
8	GO	0.040	110	15	27
9	Material II	0.040	110	15	38
10	Material III	0.040	110	15	42

a Isolated yield.

After attaining the optimized conditions, the domain and performance of the nanocatalyst for the construction of *N*,*N*′-alkylidene bisamides were assessed through usage of miscellaneous aromatic aldehydes (carrying diverse electron-attracting and electron-releasing substituents on their *ortho*, *meta* or *para* positions), and also aromatic and aliphatic amides in the reaction; the gained results are reported in Table 4. It was found that all substrates afforded the relevant bisamides in short times and high to excellent yields; these results confirmed wide domain and high efficiency of NGFRNH to catalyze the reaction.

**Table 4:** The construction of various derivatives of *N*,*N*′-alkylidene bisamide using NGFRNH

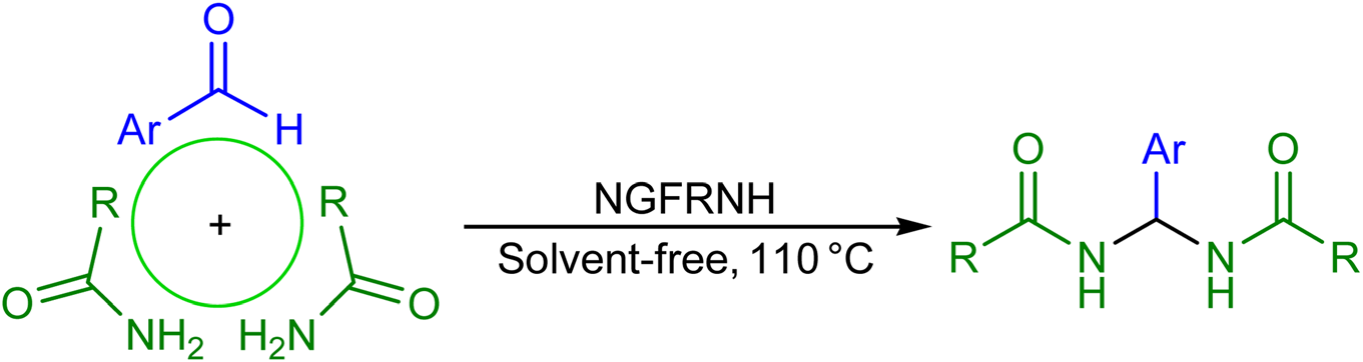					
Product no.	Ar	R	Time (min)	Yield^a^ (%)	M.p. (°C) [lit.]
1	C_6_H_5_	C_6_H_5_	20	94	222–225 (220–221)^45^
2	2-O_2_NC_6_H_4_	C_6_H_5_	20	93	255–257 (257–259)^41^
3	3-O_2_NC_6_H_4_	C_6_H_5_	25	97	226–228 (228–230)^47^
4	4-O_2_NC_6_H_4_	C_6_H_5_	20	96	260–262 (261–263)^42^
5	2-ClC_6_H_4_	C_6_H_5_	15	97	243–245 (242–244)^47^
6	4-ClC_6_H_4_	C_6_H_5_	15	98	256–259 (258–261)^43^
7	2,4-Cl_2_C_6_H_3_	C_6_H_5_	25	97	203–205 (201–203)^44^
8	4-Cl,3-O_2_NC_6_H_3_	C_6_H_5_	25	94	247–249 (250–252)^38^
9	4-BrC_6_H_4_	C_6_H_5_	15	97	254–257 (252–254)^42^
10	4-FC_6_H_4_	C_6_H_5_	15	97	230–233 (227–229)^44^
11	4-MeOC_6_H_4_	C_6_H_5_	30	89	220–222 (223–225)^44^
12	4-MeC_6_H_4_	C_6_H_5_	15	95	241–244 (241–244)^43^
13	4-O_2_NC_6_H_4_	CH_3_	25	97	257–260 (260–265)^39^
14	4-ClC_6_H_4_	CH_3_	15	96	254–257 (252–255)^38^
15	4-MeOC_6_H_4_	CH_3_	20	92	213–215 (215–217)^39^

a Isolated yield.

**Scheme 3:**
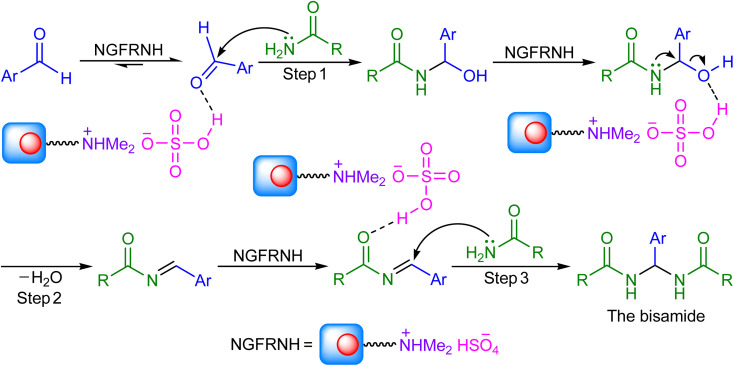
The mechanism.

On basis of the literature,^42,43,47^ a reasonable mechanism was suggested for the construction of *N*,*N*′-alkylidene bisamides (Scheme 3). Nano-[GO@Fe_3_O_4_@R-NHMe_2_][HSO_4_] can catalyze the reaction by its acidic group (hydrogen sulfate); its roles involve: (i) activating the electrophiles in steps 1 and 3 to accept nucleophilic attack of amide, and (ii) conversion of the hydroxyl group to a good leaving group in step 2 for elimination of a H_2_O molecule.

**Fig. 8:**
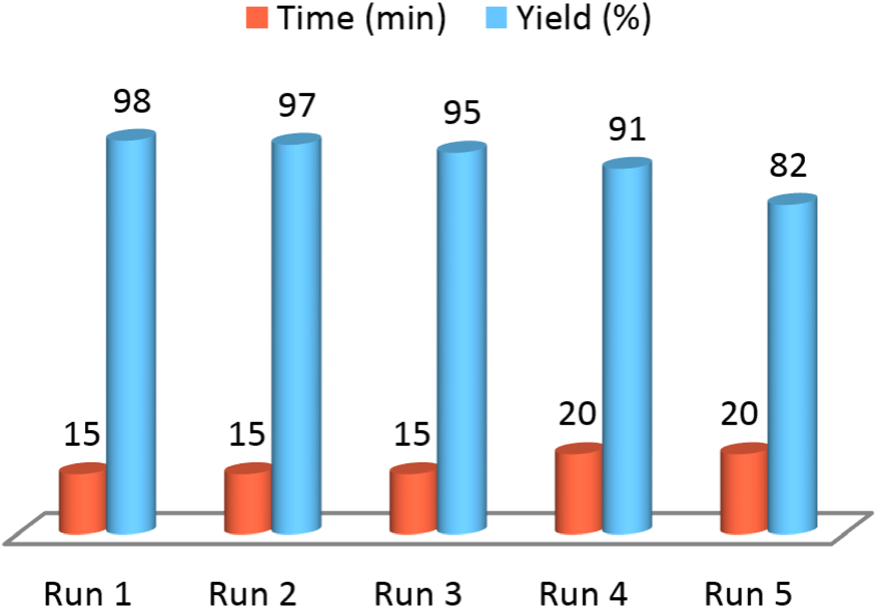
The recoverability results of NGFRNH.

Capability of NGFRNH for recovering and reusing was perused on the reaction of 4-chlorobenzaldehyde and benzamide (Scheme 2); it was recovered pursuant to the described way in experimental section, and reused for three times without remarkable loss of catalytic activity (Fig. 8). However, in fourth recycling (run 5), the reaction yield was significantly decreased.

To compare NGFRNH with the reported catalysts, the construction of bisamides 1, 6 and 12 was chosen, and the catalysts were compared in terms the reaction conditions, time and yield; Table 5 illustrates this comparison. The reaction yields of our catalyst are higher than the reported ones, and the reaction times are shorter than most of the reported catalysts showed in Table 5. The reaction conditions of NGFRNH are better than some catalysts, and are same with the others (in terms of performing the reaction under solvent-free conditions or in solvent). The reaction temperature of NGFRNH is as same as some catalysts, and is higher than the others. The another advantage of NGFRNH with respect to some catalysts reported in Table 5 is ability to catalyze the reaction in the case of aromatic and aliphatic amides.

**Table 5:** The construction of bisamides 1, 6 and 12 using NGFRNH and some reported catalysts

Catalyst	Conditions	Time (min) for products 1/6/12	Yield (%) for products 1/6/12	Ref.
NGFRNH	Solvent-free, 110 °C	20/15/15	94/98/95	This work
Nano-[Mn-PSMP]Cl_2_^a^	EtOH, reflux	—b/150/270	—b/85/75	38
Ph_3_CCl	EtOH, 60 °C	—b/35/—b	—b/90/—b	39
HPVAC-20^c^	Solvent-free, 110 °C	35/25/40	93/96/90	40
Montmorillonite K10	Solvent-free, 100 °C	80/—b/—b	85/—b/—b	41
GO@Gl-SO_3_H^d^	Solvent-free, 110 °C	15/10/15	91/96/90	42
3D-network polymer supported ionic liquid	Toluene, reflux	30/25/30	85/83/87	43
Nano-[DSPECDA][HSO_4_]^e^	Solvent-free, 90 °C	30/—b/30	91/—b/79	44
ZnO/KIT-6@NiFe_2_O_4_	Solvent-free, 60 °C	10/10/10	90/94/75	45
C/TiO_2_–SO_3_H	Solvent-free, 100 °C	90/120/120	93/93/90	46
KH_2_PO_4_ supported on silica	Solvent-free, 80 °C	15/15/15	87/90/71	47

a Nano-Mn-[phenyl-salicylaldimine-methyl-pyranopyrimidinedione]Cl_2_.

b In the research, this product has not been constructed.

c H_5_[PV_2_W_10_O_40_] immobilized on clay.

d GO grafted with SO_3_H-functionalized glycerin.

e Nano-2-[*N*′,*N*′-dimethyl-*N*′-(silica-*n*-propyl)ethanaminium chloride]-*N*,*N*-dimethylaminium bisulfate.

## 4. Conclusions

Briefly, we have fabricated a novel graphene oxide-based magnetic nanocomposite possessing an acidic group (HSO_4_^−^); it may catalyze organic transformations which require acidic catalyst to carry out. In this research, we have successfully applied the nanocomposite as catalyst to construct *N*,*N*′-alkylidene bisamides from aryl aldehydes (1 eq.) and primary amides (2 eq.); the privileges of this approach comprise wide domain, high performance, construction of the products in short times and excellent yields, efficiency of the protocol to fabricate the bisamides from aromatic and aliphatic amides, utilization of solvent-free conditions, magnetically recovering the catalyst, recoverability of the catalyst for three times without significant loss of its activity and good accordance with principles of green chemistry.

## Data availability

The data supporting this article have been included as part of the ESI.†

## Author contributions

Abdolkarim Zare: investigation, project administration, supervision, formal analysis, writing – original draft, writing – review & editing. Marziyeh Barzegar: methodology, formal analysis, writing – original draft. Esmael Rostami: investigation, supervision, formal analysis. Ahmad Reza Moosavi-Zare: investigation, writing – review & editing, formal analysis.

## Conflicts of interest

There are no conflicts to mention.

## Supplementary Material

RA-014-D4RA04136D-s001
