# Supplementary material for: Fabrication of a novel graphene oxide based magnetic nanocomposite and its usage as a highly effectual catalyst for the construction of N,N′-alkylidene bisamides
Source: RSC Adv. 2024 Aug 13;14(35):25235–46. doi: 10.1039/d4ra04136d (PMC11320193; doi:10.1039/d4ra04136d)
Supplement: RA-014-D4RA04136D-s001 [file RA-014-D4RA04136D-s001.pdf]

Supplementary Information (SI) for RSC Advances.
This journal is © The Royal Society of Chemistry 2024

Supplementary information

Fabrication of a novel graphene oxide based magnetic nanocomposite and its usage as a highly effectual catalyst for the construction of *N*,*N′*-alkylidene bisamides

Abdolkarim Zare,*[a] Marziyeh Barzegar,*[b] Esmael Rostami[b] and Ahmad Reza Moosavi-Zare[c,d]

[a]*Department of Chemistry, Faculty of Nano and Bio Science and Technology, Persian Gulf University, Bushehr 75169, Iran. E-mail: a.zare@pgu.ac.ir, abdolkarimzare@yahoo.com*

[b]*Department of Chemistry, Payame Noor University, PO Box 19395-4697, Tehran, Iran. E-mail: barzegar.marziyeh@yahoo.com*

[c]*Department of Chemical Engineering, Hamedan University of Technology, Hamedan, 65155, Iran*

[d]*Chemistry Department, College of Sciences, Shiraz University, Shiraz 71946-84795, Iran*

Table of contents ……………………………………………………………………………. **Page**

Materials and instruments ………………..……………………………….………………… S3

Selected original spectrums of the constructed bisamides ………..……………..…..…..…S4

Figure S1. The ^{1}H NMR spectrum of bisamide **3** ……………………………..……......… S4

Figure S2. The ^{13}C NMR spectrum of bisamide **3**……………..……..……......…..…… S5

Figure S3. The ^{1}H NMR spectrum of bisamide **8**…….……………………...…………....… S6

Figure S4. The ^{13}C NMR spectrum of bisamide **8**……………………………….…...…... S7

Figure S5. The mass spectrum of bisamide **8** ……………….…………………..…...….... S8

Figure S6. The ^{1}H NMR spectrum of bisamide **10**……………………………………..... S9

Figure S7. The ^{13}C NMR spectrum of bisamide **10** …………..………………......……….. S10

Materials and instruments

All reactants and solvents were purchased from Fluka or Sigma-Aldrich Chemical Companies. Progress of the reactions was monitored by TLC using silica gel SIL G/UV 254 plates. To measure the melting points, a Thermo Scientific 9200 apparatus was used. For recording the FT-IR spectra, a Thermo device (model AVATAR) was used. The NMR spectra were recorded on a Bruker Avance DPX FT-NMR spectrometer. EDX and elemental mapping analyses were done using a TESCAN device, model MIRA II. FE-SEM instrument TESCAN (model MIRA III) was utilized for determining sizes and morphologies of the particles. VSM analysis was performed using a MDK device (Meghnatis Daghigh Kavir, Iran) at room temperature. XRD analysis was carried out by a PHILIPS apparatus (Cu Kα radiation, λ=1.54056 Å, model PW1730). TGA was done using TA apparatus (model Q600), at 25-600 ºC, with temperature increase rate of 10 $^{o}C.min^{-1}$ in argon atmosphere.

Selected original spectrums of the constructed bisamides

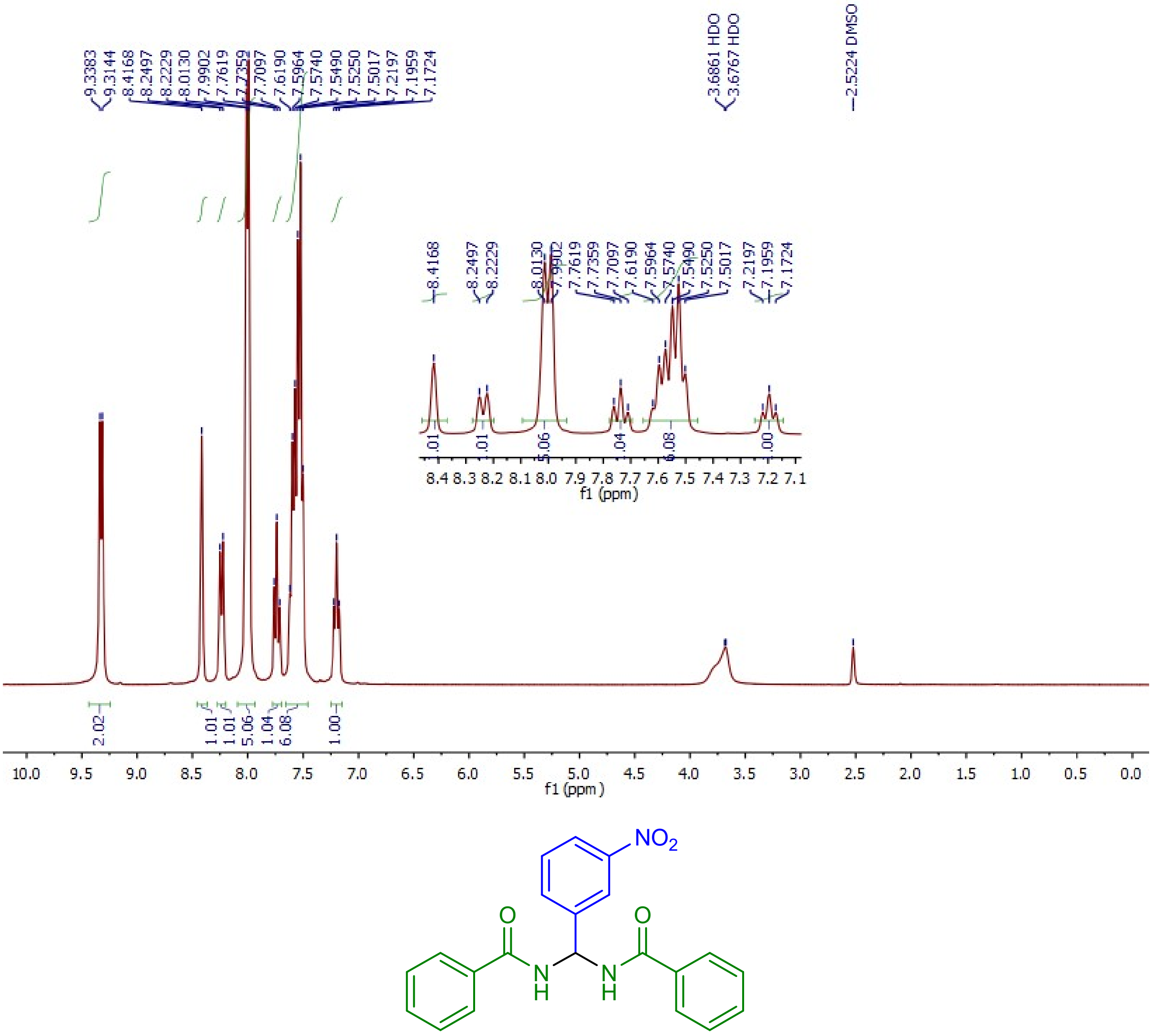

Figure S1. The ^{1}H NMR spectrum of bisamide **3**.

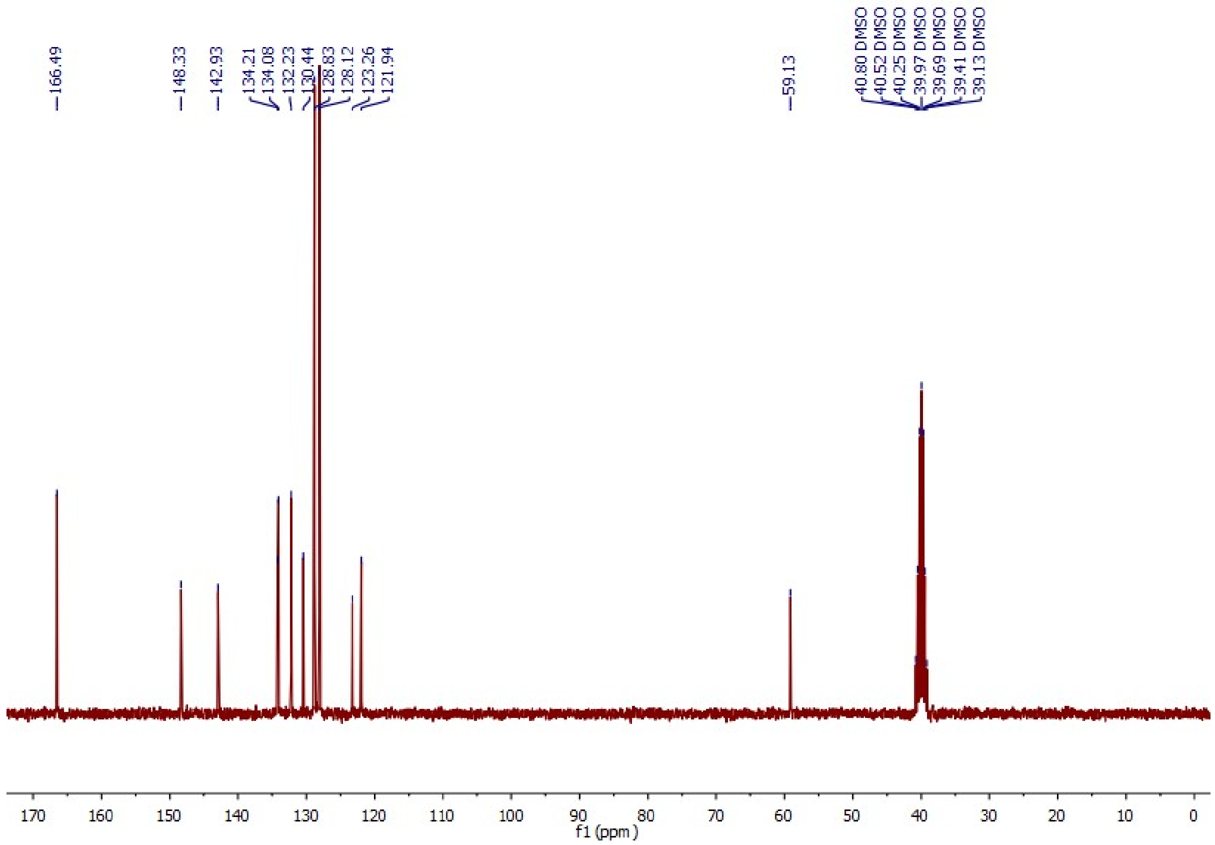

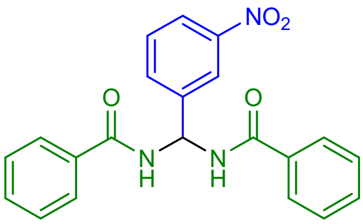

Figure S2. The ^{13}C NMR spectrum of bisamide **3**.

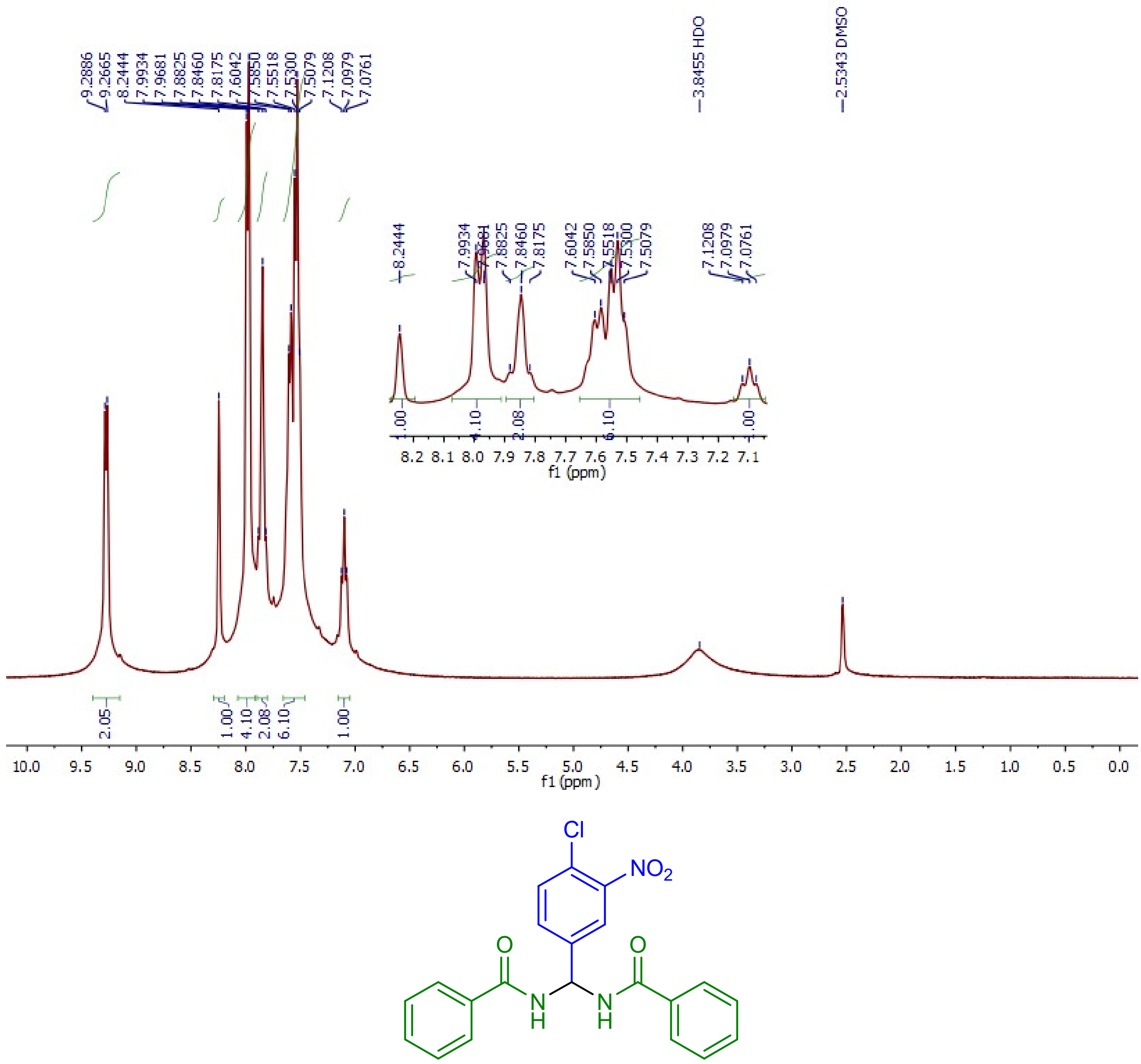

Figure S3. The ^{1}H NMR spectrum of bisamide **8**.

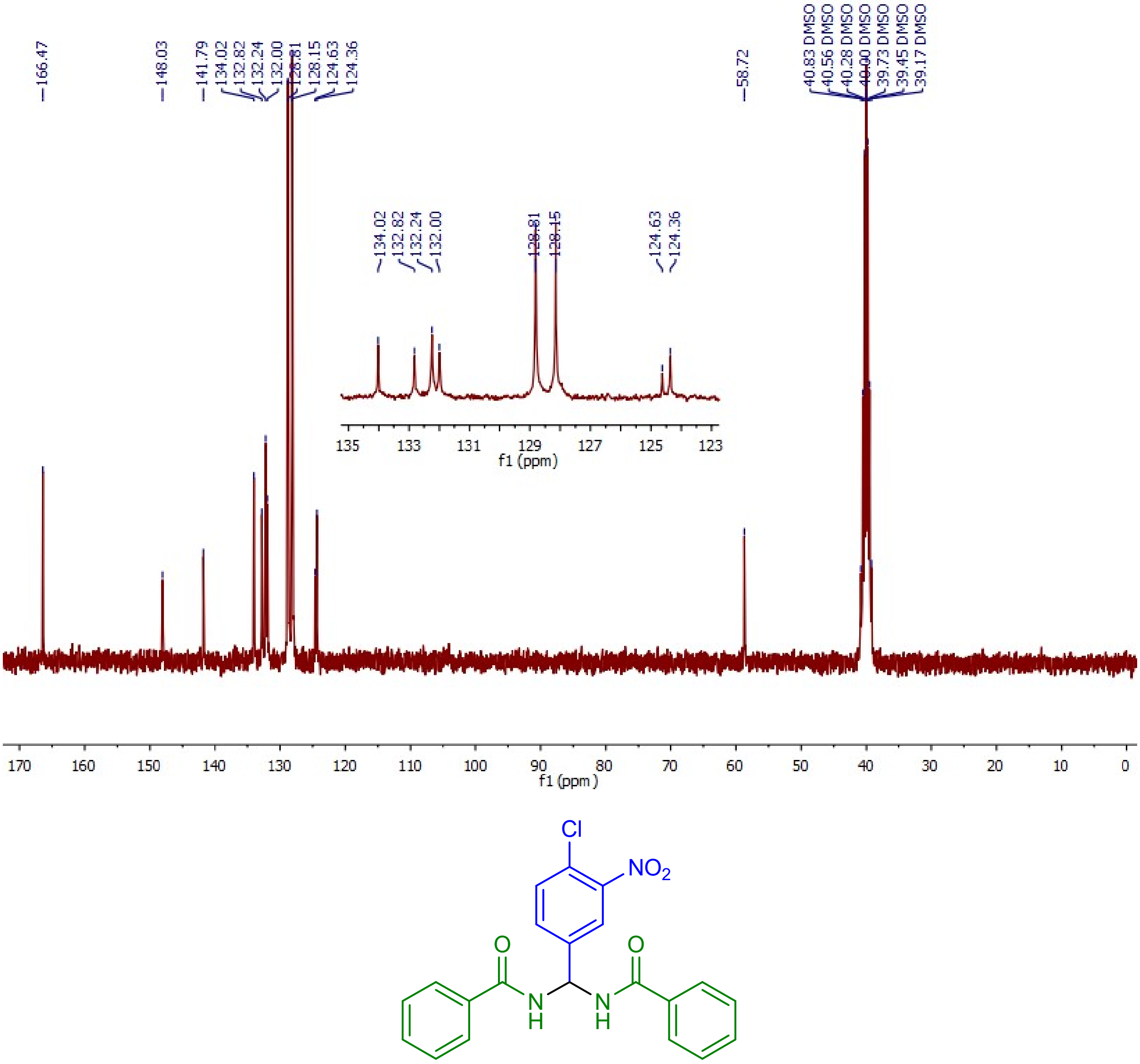

Figure S4. The ^{13}C NMR spectrum of bisamide **8**.

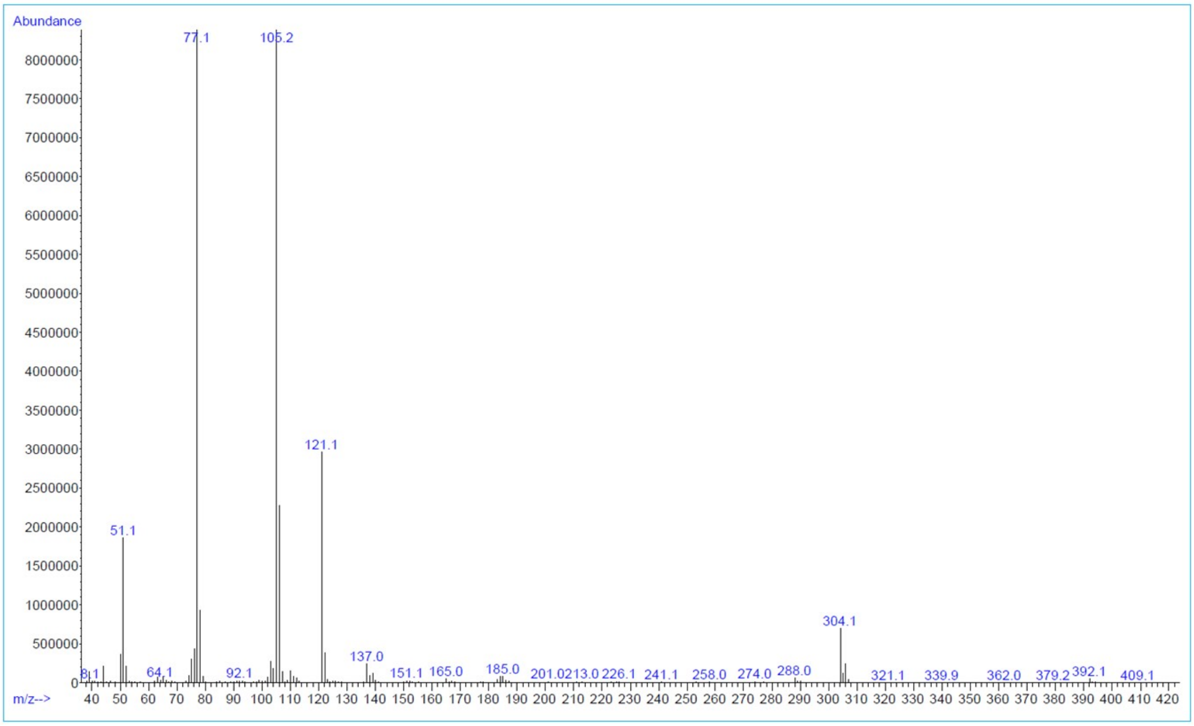

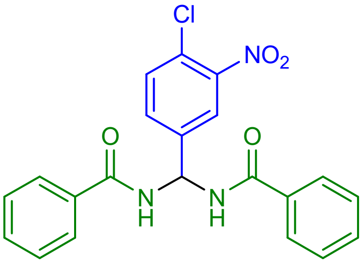

Figure S5. The mass spectrum of bisamide **8**.

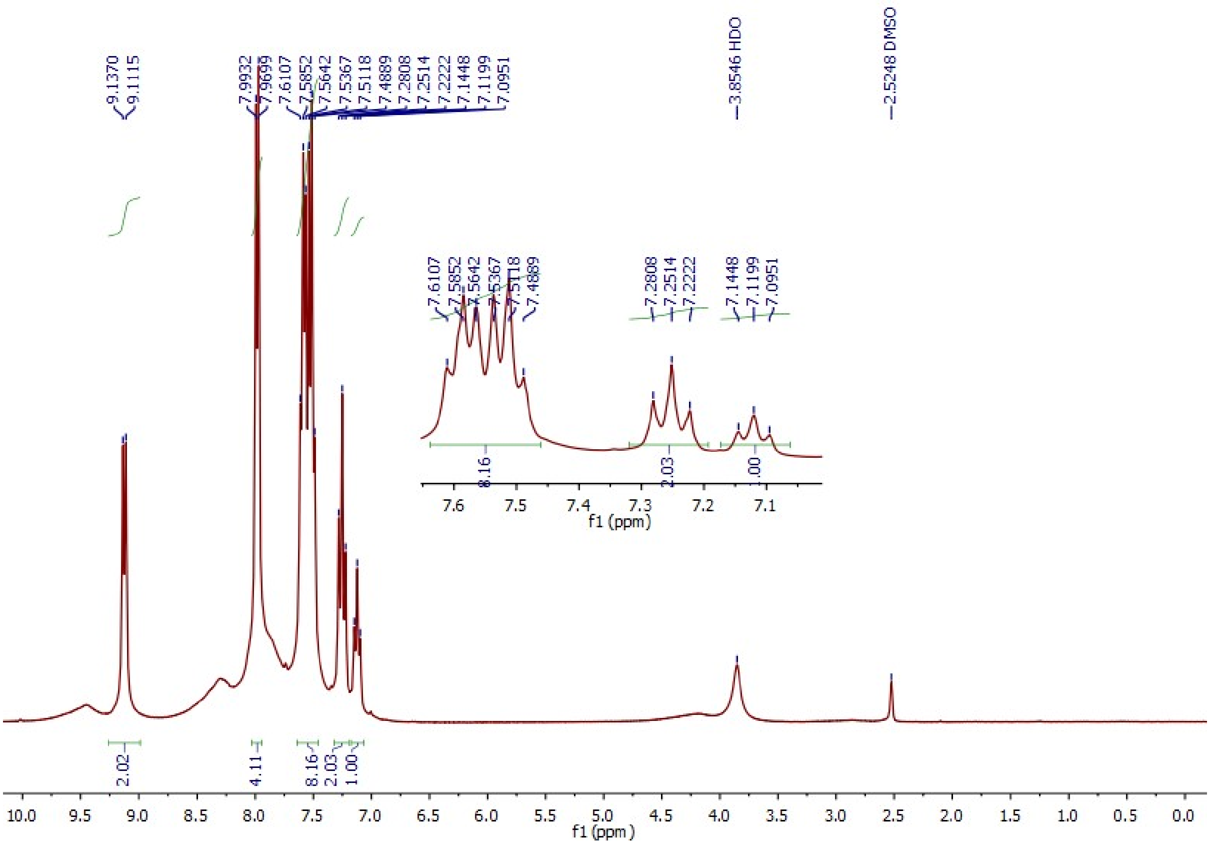

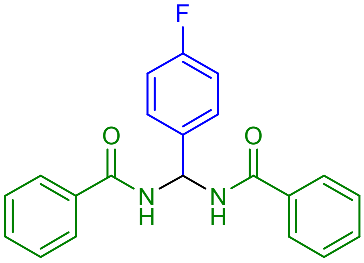

Figure S6. The ^{1}H NMR spectrum of bisamide **10**.

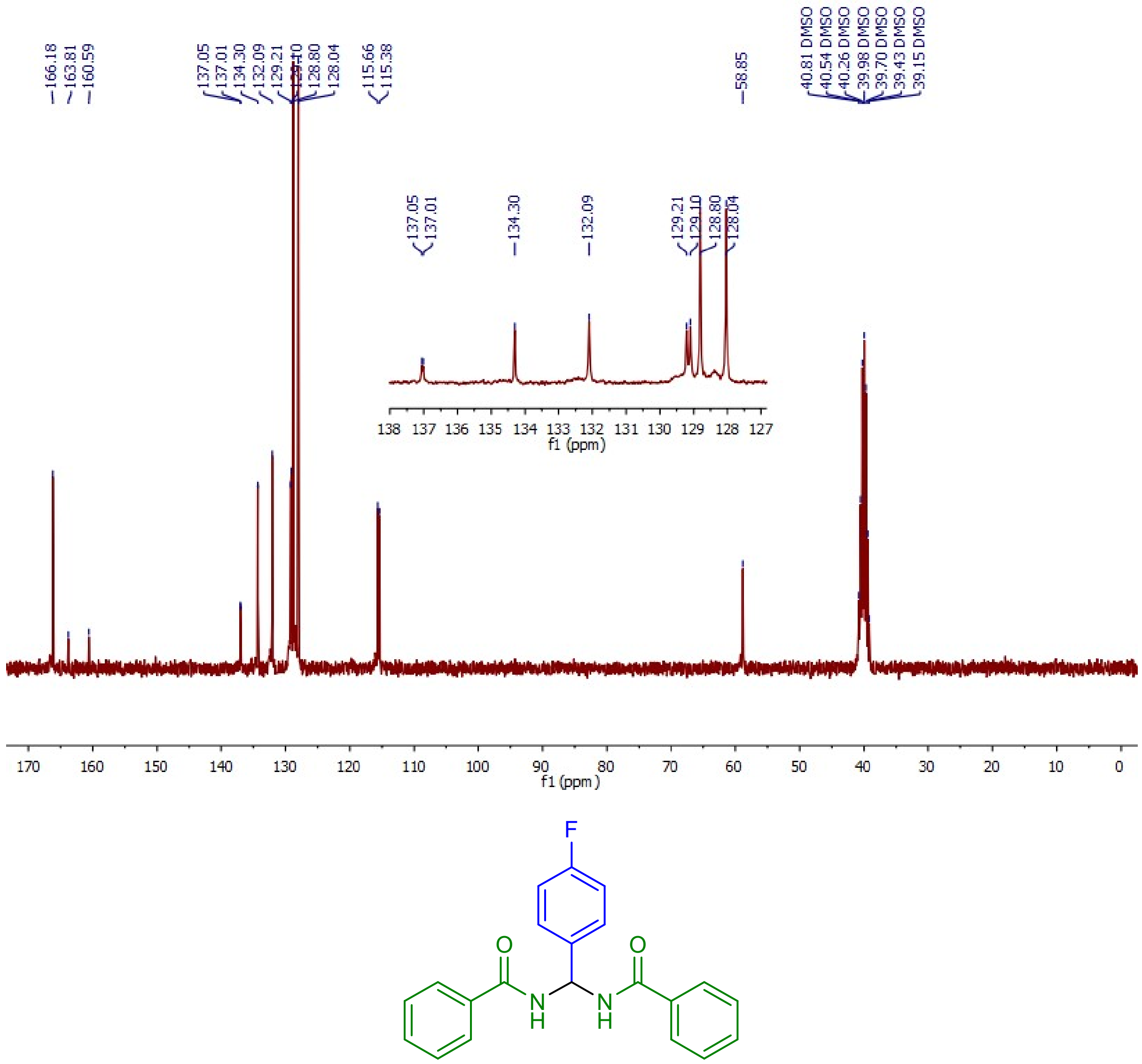

Figure S7. The ^{13}C NMR spectrum of bisamide **10**.
